# The potential of bacteriocins and bacteriophages to control bacterial disease of crops with a focus on *Xanthomonas* spp.

**DOI:** 10.1080/03036758.2024.2345315

**Published:** 2024-05-23

**Authors:** Shannon F. Greer, Mojgan Rabiey, David J. Studholme, Murray Grant

**Affiliations:** a School of Life Sciences, University of Warwick, Innovation Campus, Stratford-upon-Avon, UK; b School of Life Sciences, University of Warwick, Gibbet Hill Campus, Coventry, UK; c Biosciences, Geoffrey Pope Building, University of Exeter, Exeter, UK

**Keywords:** Antimicrobial, bacterial crop diseases, bacterial phytopathogens, bacteriocin, bacteriophage, *Xanthomonas*

## Abstract

Crop production plays a crucial role in ensuring global food security and maintaining economic stability. The presence of bacterial phytopathogens, particularly *Xanthomonas* species (a key focus of this review), poses significant threats to crops, leading to substantial economic losses. Current control strategies, such as the use of chemicals and antibiotics, face challenges such as environmental impact and the development of antimicrobial resistance. This review discusses the potential of bacteriocins, bacterial-derived proteinaceous antimicrobials and bacteriophages, viruses that target bacteria as sustainable alternatives for effectively managing *Xanthomonas* diseases. We focus on the diversity of bacteriocins found within xanthomonads by identifying and predicting the structures of candidate bacteriocin genes from publicly available genome sequences using BAGEL4 and AlphaFold. Harnessing the power of bacteriocins and bacteriophages has great potential as an eco-friendly and sustainable approach for precision control of *Xanthomonas* diseases in agriculture. However, realising the full potential of these natural antimicrobials requires continued research, field trials and collaboration among scientists, regulators and farmers. This collective effort is crucial to establishing these alternatives as promising substitutes for traditional disease management methods.

## Introduction

### Importance of crop production

Crops are cultivated not only for food, but also for animal feed, oil and fibre, and are a source of subsistence and livelihood for many of the world’s population. Ultimately, crops underpin the food security and nutritional health of the population and contribute to the global economy. Agriculture takes up 38% of the world’s land surface (∼5 billion hectares), with one third of this being used for crop production and the remainder for livestock grazing (FAO 2020). The world population was estimated at 8 billion in November 2022 (United Nations 2022) and is predicted to reach nearly 10 billion by 2050. Currently there is a huge shortfall between production and food demand, and it is argued that present year-on-year crop yield increases gained through breeding are insufficient to meet this demand (Ray et al. 2013), although other predictions differ (Hunter et al. 2017). Thus, numerous abiotic and biotic factors impact crop production, including: climate change, soil health, water availability, weeds and pests and disease, the latter of which can cause crop losses of 20%–40% in fields and post-harvest (Savary et al. 2012). These will be further exacerbated by climate change. While chemical solutions are available for a range of pests and pathogens, management of phytobacterial diseases is much more challenging. This review will focus on the impact of bacterial diseases on crop production and discuss potential nature-driven solutions for their control.

### Bacterial outbreaks threaten crop production

Bacterial phytopathogens are responsible for numerous destructive crop diseases, which globally can have a significant economic and societal impact, particularly in developing countries. There are over 25 genera and 200 species of bacteria that are pathogenic on plants (Sharma et al. 2022), many causing devastating outbreaks and substantial crop losses. The most damaging species belong to the genera *Xanthomonas*, *Pseudomonas*, *Erwinia*, *Xylella*, *Ralstonia*, *Pectobacterium*, *Pantoea*, *Agrobacterium*, *Burkholderia*, *Acidovorax*, *Clavibacter* and *Streptomyces* (Sharma et al. 2023). Bacterial plant infections can present as rots, cankers, galls, wilts and leaf and fruit spots which can seriously impact both yield and quality.

**Figure 1.**
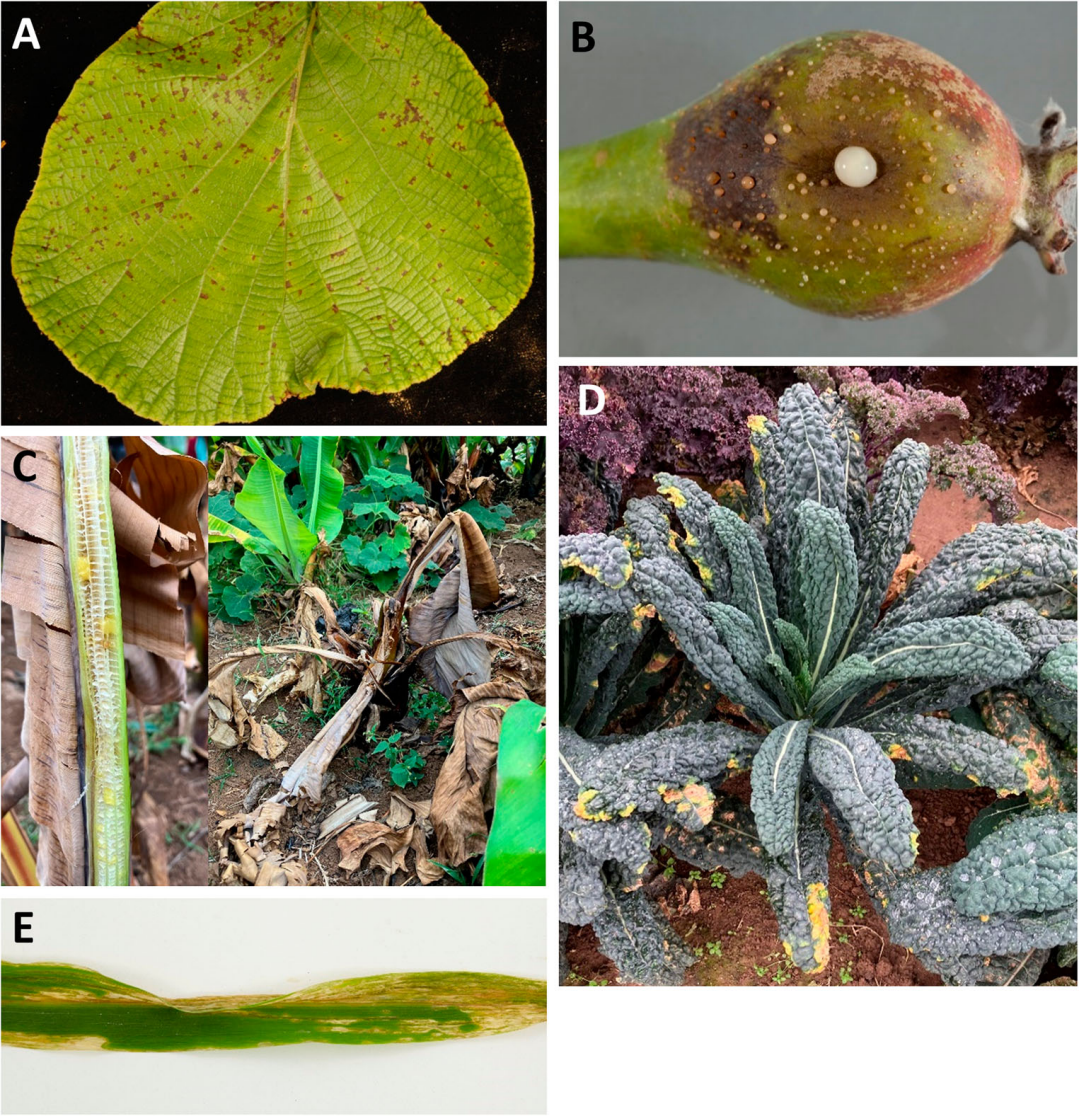
Phytobacterial diseases of crops. **A**, Leaf spot on kiwi (hort16A) caused by *Pseudomonas syringae* pv. *actinidiae.* Image credit: Dr. Kerry Everett (Plant & Food Research, New Zealand) **B**, Fire blight on pear caused by *Erwinia amylovora*. Image credit: Brian Carter and David Crossley (Copyright of Fera Science, UK) **C**, Banana *Xanthomonas* wilt on enset caused by *Xanthomonas vasicola* pv. *musacearum.* **D**, Black rot of brassicas (cavolo nero) caused *by Xanthomonas campestris* pv. *campestris*. **E**, Leaf streak of maize caused by *Xanthomonas vasicola* pv. *vasculorum*. Image credit: Adam Bryning and David Crossley (Copyright of Fera Science, UK).

In recent times, several phytobacterial outbreaks have occurred on a newsworthy scale. For example, bacterial kiwifruit canker caused by *Pseudomonas syringae* pv. *actinidiae* (*Psa*) causes symptoms like dieback, cankers, leaf spots and gum exudation (Figure 1A). It has blighted kiwifruit production in New Zealand orchards since 2010, and it is expected to cost ∼NZ$900 million to implement a long-term control plan, underscoring the significant economic challenges *Psa* poses (Butler et al. 2013; Gillespie 2023). *Xylella fastidiosa*, causal agent of Olive Quick Decline Syndrome, was first reported in Italy in 2013 and has since spread through Europe. Today it poses a major threat to olive production in other regions of Europe and has a predicted economic impact in excess of €20 billion (McGrath 2020; Schneider et al. 2020). Fire blight of pome fruits like apples, pears and quinces caused by *Erwinia amylovora* (Figure 1B) can disrupt orchards for several years, with loss of an entire year’s harvest in infected orchards, like those in New Zealand’s Hawke’s Bay Region, estimated to cost ∼NZ$10 million (Zhao et al. 2019). *Ralstonia solanacearum* has a wide host range infecting solanaceous crops (potatoes, tomatoes and peppers) and causes Moko disease of musaceous crops (bananas, plantains and ensetes). On potatoes alone, it is responsible for US$1 billion losses a year, and Moko disease causes significant hardship for subsistence farmers (EPPO 2021) and threatens food and nutritional security.

### Current control methods for bacterial plant diseases are inadequate

Unlike fungal pathogens, there are limited chemical interventions available to control bacterial phytopathogens. The more effective traditional bactericides like antibiotics, Bordeaux mixture or more elaborate copper-containing nanoparticles (e.g. Kocide 3000 (Dupont, Certis)) can effectively control bacterial plant diseases. However, their use is often strictly regulated or in fact being withdrawn because of their adverse environmental impacts, toxic bioaccumulation and potential threat to human health by contributing to antimicrobial resistance (AMR) (La Torre et al. 2018; Lamichhane et al. 2018). Furthermore, resistance to copper and antibiotics is already widely reported in plant pathogenic bacteria like *E. amylovora* (Thomson et al. 1993) and *Psa* (Nakajima et al. 2002; Colombi et al. 2017; Aono et al. 2024) and is particularly prevalent in xanthomonads (Voloudakis et al. 2005; Behlau et al. 2011, 2020; Areas et al. 2018; Sundin and Wang 2018). Other control practices include using pathogen-free seed or propagation material, growing resistant crop varieties, sanitising farm tools and machinery, rotating crops to prevent disease build-up in the field and destroying infected material by burning. The latter may require destruction of an entire crop, something that is simply not an option for subsistence farmers. Therefore, alternative, sustainable biocontrols for bacterial plant diseases are desirable.

In this review, we will focus specifically on the highly destructive *Xanthomonas* species, examining challenges of current control methods and focusing on the potential recent advances in the use of bacteriocins and bacteriophages.

### *Xanthomonas* diseases devastate a broad range of crops

Xanthomonads are vascular or non-vascular Gram-negative bacteria, which can infect both monocots and dicots. Species of the *Xanthomonas* genus are particularly damaging, causing disease on over 400 plant hosts, sometimes resulting in complete losses of important food and economically essential crops like rice, cereals, beans, cassavas, brassicas and bananas (Timilsina et al. 2020) (Figure 1) (Table 1). The genus currently comprises 33 species (Parte et al. 2020; LPSN 2024), many of which are further subdivided into pathovars and races that have historically been determined based on their host range, although genomic sequence data is now informing many taxonomic revisions (Constantin et al. 2016; Timilsina et al. 2019; Studholme et al. 2020; Harrison et al. 2023).

**Table 1.** Economically important *Xanthomonas* species and their hosts and associated yield losses (where known).

*Xanthomonas* pathogen	Host	Yield loss	Reference
*X. oryzae* pv. *oryzae*	Rice	≤70%	Niones et al. (2022)
*X. vasicola* pv. *musacearum*	Banana and enset	≤100%	Blomme et al. (2023), Kikulwe et al. (2019)
*X. vasicola* pv. *vasculorum*	Maize and sugar cane	<15% 30%–40%	Ortiz-Castro et al. (2020) Hartman (2018)
*X. cassavae X. phaseoli* pv. *manihotis*	Cassava	<90%	Zárate-Chaves et al. (2021)
*X. hortorum* pv. *gardneri X. euvesicatoria* pv*. perforans X. euvesicatoria* pv. *euvesicatoria X. vesicatoria*	Tomato	≤50%	Horvath et al. (2012)
*X. cucurbitae*	Pumpkin	≤90%	Babadoost and Ravanlou (2012)
*X. phaseoli* pv. *phaseoli*	Bean	≤75%	Foucher et al. (2020) CABI (2019)
*X. campestris* pv. *campestris*	Brassicas	>50%	Singh et al. (2011)
*X. translucens*	Cereals	≤40%	McMullen and Adhikari (2011)
*X. citri* pv. *citri*	Citrus	≤50%	Ali et al. (2023)
*X. hortorum* pv*. vitians*	Lettuce	≤100%	Toussaint (1999)

Bacterial blight of rice caused by *X. oryzae* pv. *oryzae* (*Xoo*) is of particular concern, and severe infections can reduce yield by as much as 70% in Asia (Niones et al. 2022) (Table 1). *Xoo* poses a serious threat to global food security, as rice is a staple food for more than half the world’s population and is the most valuable crop, with a gross production value of US$312 billion (FAO 2021). Also less well known, banana *Xanthomonas* wilt (BXW) (Figure 1C) caused by *X. vasicola* pv. *musacearum* is another recent threat, impacting the livelihoods and food security of millions of Africans for whom banana is a major calorific dietary component. It was first identified on Ethiopia’s *Ensete ventricosum*, an orphan staple food crop for >20 million in the country (Koch et al. 2022) by Yirgou and Bradbury (1968). Now, 70%–100% of enset farms in Ethiopia have been blighted by BXW (Blomme et al. 2023), where it can cause total crop losses (Table 1). In the early 2000s, it spread to banana plantations in other East African countries including Uganda, Rwanda, Tanzania and Democratic Republic of Congo (Nakato et al. 2021). One of the most widespread *Xanthomonas* diseases is black rot of brassicas, caused by *Xanthomonas campestris* pv. *campestris* (*Xcc*) (Figure 1D) and is present in 93 countries and 6 continents (CABI 2022). A global average of 89.7 million tonnes of vegetable brassicas (cabbage, cauliflower and broccoli) are produced annually, with an estimated value of UD$32.3 billion in 2020 (Greer et al. 2023; FAO 2023a, 2023b). *Xcc* poses a significant threat to vegetable production worldwide, as it can cause yield losses of >50% in susceptible varieties (Singh et al. 2011) (Table 1). Another emerging threat is that of *X. vasicola* pv. *vasculorum* (*Xvv*), the causal agent of bacterial leaf streak of maize (Figure 1E), and sugarcane gumming disease. This disease was first reported in South Africa by Dyer (1949), where it remained with limited impact for decades before spreading to South America in 2010 and breaking out in major maize growing states of the United States from 2016 (Triplett and Patel 2020). Given the recent outbreaks of *Xvv*, it is still not clear what its yield impact is on maize, but some have reported ∼15% yield losses (Ortiz-Castro et al. 2020), and in sugarcane *Xvv* has been reported to cause 30%–40% reduction in plant tonnage when systemic infection is achieved (Hartman 2018) (Table 1).

Biocontrol of *Xanthomonas* diseases has attracted considerable research (Marin et al. 2019). Despite this, there are very few commercially available products. Research for disease control has predominantly focussed on the use of bacteriophages (Nakayinga et al. 2021) or antagonistic bacteria like *Bacillus* spp. and non-pathogenic pseudomonads (Marin et al. 2019). In comparison, research on the use of antimicrobial proteins/peptides like bacteriocins as plant protection products is notably limited despite these being widely used in food preservation (Negash and Tsehai 2020). Generally, studies only investigate the activity of single biocontrol agents and, as seen for copper-based products and antibiotics, such overreliance on a single bioactive risks development of AMR. This review will critically evaluate the potential of largely unexplored bacteriocins, bacteriophages and, importantly, feasibility of dual applications for the control of *Xanthomonas* diseases.

## Bacteriophages

**Figure 2.**
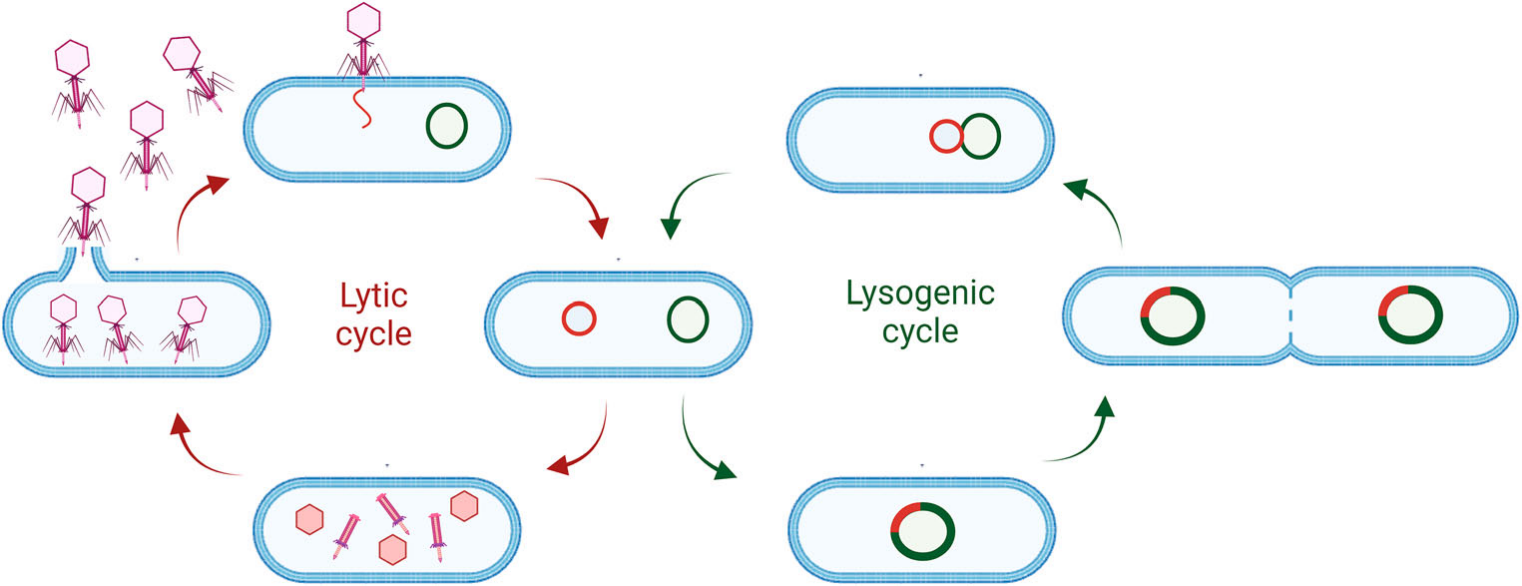
Following phage infection of a bacterial cell, phages exhibit two distinctive life cycles: lytic and lysogenic. During the lytic cycle, phages hijack bacterial cell machinery to produce progeny phages, eventually leading to bacterial lysis. The lysogenic cycle leads to integration of phage DNA into bacterial chromosomes which can be inherited by daughter cells. Under certain environmental conditions a phage may switch from the lysogenic to the lytic cycle. Virulent phages only undergo the lytic cycle. Created in Biorender.com.

A bacteriophage, often referred to simply as a phage, is a virus that specifically infects and replicates within bacterial cells. Phages are highly specific to their host (Grace et al. 2021). They are used in biotechnology, and extensive research is ongoing to use phages for controlling bacterial diseases. Phages can exhibit two distinctive life cycles upon infection of a bacterial cell: lytic and lysogenic (Figure 2) (Clokie et al. 2011; Fortier and Sekulovic 2013). When a lytic phage infects a bacterial cell, it immediately begins replicating within the cell by hijacking the host replication machinery, leading to the production of numerous phage progeny, lysing the cell and releasing new phage particles, which can then infect other bacterial cells. Lytic phages are responsible for the rapid destruction of the host bacterial population. When a lysogenic phage infects a bacterial cell, it integrates its genetic material into the host’s genome. This integrated genetic material is called a prophage. Under certain conditions, such as environmental stress, the prophage can excise from the bacterial genome and switch to a lytic life cycle, and by doing so can lead to horizontal gene transfer (HGT) (Hulin et al. 2023). Lytic phages are often preferred for rapid reduction of pathogen populations given their bacterial host specificity, which minimises damage to other non-target bacteria with no risk of HGT.

Most phages isolated for biocontrol purposes belong to the lytic phages of the *Caudovirales* order. *Caudovirales* families (*Podo*-, *Myo*- and *Siphoviridae*), are attractive for biocontrol purposes due to their diversity, host specificity and lytic capabilities, which can help manage bacterial pathogens in agricultural settings (Villalpando-Aguilar et al. 2023). While *Caudovirales* are commonly used, other phage orders and families can also be explored for specific biocontrol applications based on the target pathogen and environmental conditions.

### Current uses

Phages have been studied for their ability to target and control bacterial pathogens that affect humans, animals and plants (Rogovski et al. 2021). Currently in the United States there are a few commercial phage biocontrol products for agricultural application available. These include AgriPhage-Fire Blight against apple blight of apple and pear caused by *E. amylovora*; Agriphage-XCV and PST against tomato and pepper bacterial spots caused by *X. campestris* pv. *vesicatoria* and *P. syringae* pv. *tomato*; Agriphage-CMM against tomato bacterial canker caused by *Clavibacter michiganensis* pv. *michiganensis*; Agriphage-Citruc Canker against bacterial citrus canker caused by *X. citri* pv. *citri* and Agriphage-Nut and Stone Fruit against peach bacterial spots caused by *X. arboricola* pv. *pruni*; cherry canker caused by *P. syringae* pv. *syringae*; and almond blast, walnut blight and hazelnut blight caused by *X. arboricola* pv. *pruni*, *X. arboricola* pv. *juglandis* and *P. syringae* pv*. syringae*, respectively (OmniLytics 2023). These phage products are formulated with specific lytic phages that can infect and reduce the population of the pathogens in orchards. The specific phages in AgriPhage products are proprietary (Grace et al. 2021; Nakayinga et al. 2021), preventing genomic mining of related phages in databases. Erwiphage is currently being used in Hungary to treat fire blight of apple and pear (*E. amylovora*) (Erwiphage 2020).

Phages have also been explored as biocontrol agents for *P. syringae* pv. *actinidiae* on kiwifruit (Jayaraman et al. 2020; Warring et al. 2022). Integrated pest management (IPM) approaches using phages in combination with antibacterial agents, such as carvacrol, have shown promise in inhibiting *Psa* and preventing biofilm formation. Phages have been isolated and characterised for other *P. syringae* pathovars causing bacterial canker in fruit trees, such as cherry canker (Rabiey et al. 2020) and *P. syringae* pv. *aesculi*, the causal agent of bleeding canker in European horse chestnut trees (James et al. 2020).

Although less actively studied, phages have been discovered against several *Xanthomonas* species (Table 2) (see also Nakayinga et al. 2021). Most of these phages have shown exciting potential in significantly reducing the bacterial population, however, no further studies or commercialisation have been reported, and field trials are needed to evaluate their biocontrol efficacy.

**Table 2.** Phages effective against *Xanthomonas* species.

Phage	Phage species	Target	Plant disease	Reference
Xaj2 Xaj24	*Siphoviridae Podoviridae*	*X. arboricola* pv. *juglandis*	Blight of walnut	Dömötör et al. (2016)
Xp3-A Xp3-I	*- -*	*X. arboricola* pv. *pruni*	Leaf spot and cankers of *Prunus*	Civerolo (1970)
XacF1	*Inoviridae*	*X. citri* pv. *citri*	Asiatic citrus canker of citrus	Ahmad et al. (2014)
XacN1	*Myoviridae*	*X. citri* pv. *citri*	Asiatic citrus canker of citrus	Yoshikawa et al. (2018)
Φ16, Φ17A, Φ31	*Autographiviridae*	*X. euvesicatoria* pv. *allii*	Leaf blight of onions	Nga et al. (2021)
φXOF4	*Siphoviridae*	*X. oryzae* pv. *oryzae*	Leaf blight of rice	Ranjani et al. (2018)
XMP-1		*X. citri* pv. *vignaeradiatae*	Leaf spot of mungbean	Borah et al. (2000)
FoX2 FoX6	*Myoviridae*	*X. campestris* pv. *campestris*	Black rot of brassicas	Holtappels et al. (2022)
Langrundblatt 1 and2 Pffeifenkraut	*Siphoviridae*	*X. translucens* pv*. translucens*	Leaf streak of cereals	Erdrich et al. (2022)

Thus, while phages show enormous promise as a biocontrol strategy against bacterial pathogens in agriculture, further research and field trials are essential to assess their practicality and effectiveness of both delivery and longer-term persistence/survival in managing these diseases.

### Strengths and challenges

The use of phage-based products is seen as an eco-friendly and sustainable alternative to chemical treatments, particularly in organic and environmentally conscious farming practices. It offers a targeted solution for bacterial diseases while minimising the environmental impact associated with conventional pesticides. The efficacy, well-established regulatory approval processes and practicality of phage-based solutions are key factors in their broader adoption. Here we highlight some challenges in using phages as a biocontrol, but also discuss how these challenges can be overcome.

*Host range*: Phages are typically host-specific, meaning they can only target a narrow range of bacterial strains. This property makes design of phage cocktails against clonal bacterial diseases like kiwi canker caused by *Psa* (Mazzaglia et al. 2012) relatively easy but more challenging for diseases like black rot of brassicas, which is caused by multiple different races of *Xcc* (Vicente et al. 2001). However, having a broad host range need not be advantageous, as such phages might have off-target effects and impact other beneficial and commensal microbes (Loc-Carrillo and Abedon 2011).

*Emergence of phage-resistant bacteria*: Bacteria can develop resistance to phages over time, leading to a reduction in effectiveness of phage biocontrol and requiring continuous exploration for new phages, which is restrictive from both a management and an economic perspective. Although phages and bacteria engage in an ongoing evolutionary arms race, phages evolve to recognise new bacterial strains or adapt their strategies to effectively infect these ‘resistant’ bacteria. A better mechanistic understanding of these interactions will help develop more effective and sustainable strategies to combat bacterial pathogens (Hasan and Ahn 2022).

*Application challenges*: Phage application in the field can be challenging, especially for large trees or crops, as it necessitates ensuring uniform coverage and maintaining phage viability during application. However, recent advances in technology, such as advances in genetic engineering for modifying phages to target specific bacterial strains more effectively (Huss and Raman 2020), developments in formulation and delivery methods to improve phage stability and efficacy under diverse environmental conditions (Rabiey et al. 2020) and enhancements in monitoring and data analysis tools for assessing the outcomes of phage interventions in real-world settings (Villalpando-Aguilar et al. 2023), will facilitate successful application of phages in large-scale field trials (Frampton et al. 2012; Korniienko et al. 2022; Retamales et al. 2022).

*Environmental factors*: Environmental conditions, such as UV radiation, high temperatures and pH fluctuations, impact phage stability and survival, often necessitating the use of protective formulations. Formulations containing skimmed milk, flour and sucrose have been demonstrated to be effective in significantly extending phage survival, particularly under conditions of high UV light levels and elevated temperatures (Balogh et al. 2003; Żaczek et al. 2015). Such formulations that enhance the stability and persistence of phages can serve as a protective carrier medium, shielding the phages from environmental stressors and increasing their viability on plant surfaces. This can improve the overall efficacy of phage-based biocontrol strategies, allowing for better disease management in agriculture.

*Lack of regulation*: Phage biocontrol for agricultural use is often less regulated than chemical treatments, and the long-term ecological and environmental impacts are not as well understood. Ongoing scientific studies and implementation of risk assessments can ensure that phage biocontrol is a safe and effective alternative for crop protection and will be essential to ensure its responsible and sustainable integration into agricultural practices. This necessitates comprehensive monitoring of ecosystems, continual research into the phages used and establishing protocols to prevent the potential evolution of resistance, or other unintended consequences. Collaboration between scientists, regulators and farmers is crucial for establishing guidelines that balance innovation with environmental safety, securing a promising future for phage biocontrol in agriculture.

*Impact on microbiomes*: One of the key concerns about phage biocontrol is the potential impact on the beneficial host microbiome (Wang et al. 2019). This an area that warrants considerable research effort to address this knowledge gap.

Despite these challenges, phages remain a promising approach for the biocontrol of bacterial pathogens in agriculture. Continued research and development is necessary to address these limitations and expand the use of phages in sustainable disease management.

## Bacteriocins

Protein bacteriocins (hereafter referred to as bacteriocins) are ribosomally synthesised proteins or peptides produced by bacteria and archaea that kill or restrict growth of other closely related species, helping them to establish a niche in their environment (Heilbronner et al. 2021). Bacteriocins cross a multi-layered cell envelope via a mechanism involving parasitising host proteins, often those involved in nutrient and metabolite import (Grinter et al. 2016; White et al. 2017). Bacterial outer membrane proteins serve as receptors and translocators for bacteriocin binding or toxin import (Housden et al. 2013; White et al. 2017), while the bacteriocin producer is generally protected from its own toxin by an immunity protein. Colicin, named after its producing bacteria *Escherichia coli*, was the first bacteriocin to be discovered by Gratia (1925). Since then, hundreds of bacteriocins produced by many different bacterial and archaeal strains have been identified, and some have been deposited into relevant databases. For example, BACTIBASE is a database and a phylogenetic tool for the characterisation of bacteriocins produced by bacteria (Hammami et al. 2007). With increasing collections of phytobacterial genomes in public databases, it is now feasible to undertake stringent homology searches to rapidly identify and validate the activities of bacteriocins targeted against phytopathogenic bacteria. Thus, untapped potential exists to utilise diverse bacteriocins for precision targeting of bacterial phytopathogens that cause significant economic losses. Development of effective bacteriocin treatments could replace and improve on the limited and damaging chemical treatments currently in use, providing a more sustainable solution (Lamichhane et al. 2018).

Bacteriocin classification is complex due to their diversity and has changed several times as new classes have been uncovered. Classification systems differ based on combinations of different criteria such as Gram stain of the producing bacteria, molecular weight, chemistry, mode of killing and post-transcriptional modifications (PTMs). One of the simplest classification systems was proposed by Cotter et al. (2013) and places bacteriocins into two classes: Class I peptides that have undergone PTMs, which are further divided into sub-classes dependent on PTM type, and Class II heat-stable, hydrophobic peptides that have no modification where sub-classes are based on possession of certain motifs and/or structure e.g. linear vs cyclic. Together Class I and II bacteriocins are called microcins, as they have a molecular weight of <10 kDa. However, this system does not consider larger proteinaceous bacteriocins like colicins, pyocins or phage tail-like (tailocins) and lectin-like bacteriocins. Phage tail-like bacteriocins (including R- and F-type pyocins) are the largest bacteriocins, ranging in size from 20–100 kDa and can consist of up to 14 different polypeptide units (Zimina et al. 2020). Some classification systems group these larger bacteriocins in Class III and go further to define a Class IV which, are large complex bacteriocins with lipid or carbohydrate modifications (Cesa et al. 2021; Khorshidian et al. 2021). As well as being structurally diverse, bacteriocins can be functionally diverse, having broad bactericidal or bacteriostatic activity. Colicins, for example, deliver a single cytotoxic activity that can, depending on type, kill by depolarising the inner membrane, hydrolysing DNA or RNA in the cytoplasm, abolishing peptidoglycan biosynthesis or directly degrading peptidoglycan in the periplasm (Cascales et al. 2007).

### Current uses

Currently, nisin, pediocin PA-1 and Micocin® are the only FDA-approved bacteriocins and are used as food preservatives (Schofs et al. 2020; Naskar and Kim 2021). They are applied mainly to dairy products and canned food, as they give broad protection against many heat-stable, spore-forming bacterial species like *Bacillus* and *Clostridium* spp. (Negash and Tsehai 2020). Bacteriocins can be added to the food by inoculating with the producing strain or as semi-pure and pure products and are advantageous because they are colourless, odourless and tasteless. Moreover, some classes are stable at high temperatures, extreme pHs and salt concentrations (Aljohani et al. 2023). Nisin is also approved for use in veterinary medicine for treatment of intramammary infection in dairy cattle and dermatological pathologies in dogs, cats and horses (Schofs et al. 2020). Bacteriocins have also shown much promise for the treatment of human disease caused by multi-drug resistant (MDR) bacteria, although none are currently approved by regulatory agents. For example, Class II denfensins have been shown to be effective against MDR human pathogens like *Staphylococcus aureus* and *Mycobacterium tuberculosis* (Benítez-Chao et al. 2021). Colicins have been shown to kill and disrupt *Escherichia coli* biofilms, where overgrowth in the gut is associated with inflammatory conditions like Crohn’s disease (Brown et al. 2012). Colicins have also been shown to be cytotoxic to several human tumour cell types (Chumchalová and Šmarda 2003).

In comparison, only limited studies have investigated the potential of bacteriocins against phytopathogens, and none are approved as commercial products. Rooney et al. (2020b) showed that expression of putidacin L1, a lectin-like bacteriocin, in *Arabidopsis* and *Nicotiana benthamiana* gave resistance against diverse *Pseudomonas syringae* pathovars. Mirzaee et al. (2021) showed that plantaricin and leucocin bacteriocins, sourced from BACTIBASE, gave resistance to *Clavibacter michiganensis* pv. *michiganensis* and *P. syringae* pv. *tomato* when expressed in tomatoes.

There are currently no bacteriocin formulations that are suitable for direct plant application on the market. However, genomic-informed bacteriocin identification offers the opportunity to systematically screen for bacteriocins effective against specific phytobacterial diseases such as black rot of brassicas.

### Bacteriocin diversity in xanthomonads

Several studies have reported bacteriocins with antimicrobial activity in *Xanthomonas* species (Heu et al. 2001, 2005; Pham et al. 2004; Roh et al. 2008; Ghequire et al. 2012, 2018; Marutani-Hert et al. 2020). However, only one bacteriocin from *Xanthomonas*, glycinecin A from *Xanthomonas citri* pv. *glycines*, identified by Fett et al. (1987), has been researched in detail. Glycinecin A has been cloned, purified and characterised as a large (53 kDa) heterodimeric bacteriocin that is effective against other *X. campestris* pathovars and *X. oryzae* (Heu et al. 2001; Pham et al. 2004). Table 3 summarises putative *Xanthomonas* bacteriocins, including glycinecins A and R and lectin-like bacteriocins, BCN-A, B and C among others.

**Table 3.** Examples of predicted bacteriocins encoded in genomes of *Xanthomonas* species.

Bacteriocin type (reference)	UniProt accession number and references	Taxon	(Predicted) structure
Colicin (Ochiai et al. 2005; Holtsmark et al. 2008; Salzberg et al. 2008)	RefSeq: WP_200898176.1	*Xanthomonas oryzae* pv. *oryzae*	Protein not present in UniProt. No structural prediction in AlphaFold.
Glycinecin A (subunit A) (Heu et al. 2001; Pham et al. 2004)	Q93GC2	*Xanthomonas citri* pv. *glycines*	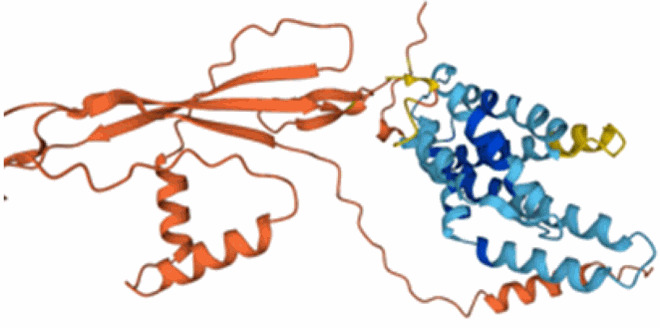
Glycinecin A (subunit B) (Heu et al. 2001; Pham et al. 2004)	Q93GC1	*Xanthomonas citri* pv. *glycines*	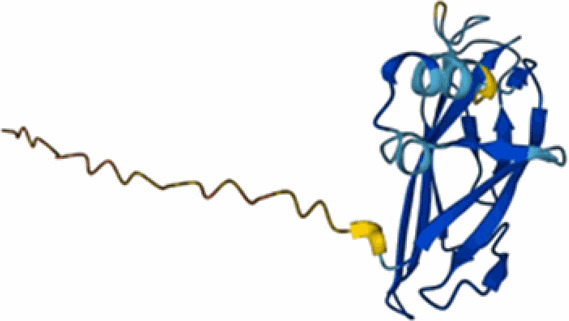
Glycinecin R (Rhs-containing) (Roh et al. 2008)	A0A7X9YTR5	*Xanthomonas citri* pv. *citri*	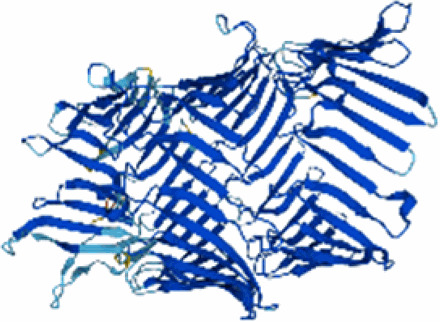
BCN-A (Rhs-containing) (Marutani-Hert et al. 2020)	UniProt: A0A0G8XA03	*Xanthomonas euvesicatoria* pv. *perforans*	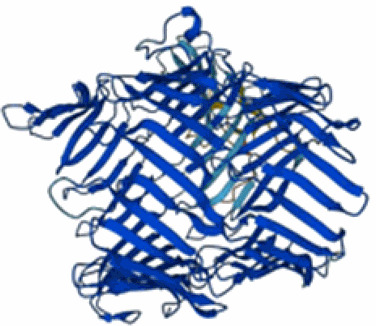
BCN-B (Marutani-Hert et al. 2020)	UniProt: B2DC89	*Xanthomonas euvesicatoria* pv. *perforans*	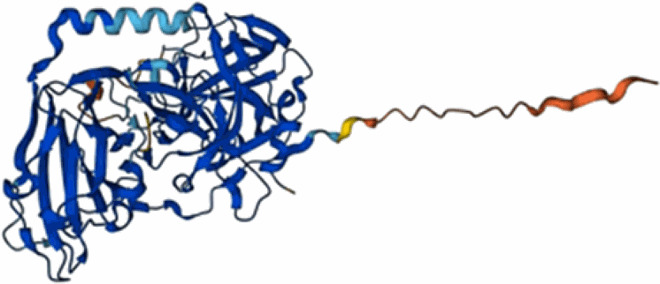
BCN-C (Marutani-Hert et al. 2020)	UniProt: B2DC92	*Xanthomonas euvesicatoria* pv. *perforans*	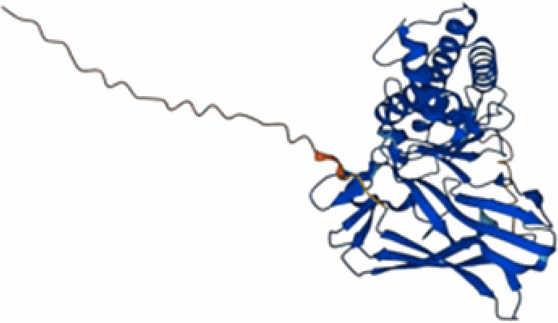
Zoocin A (M23 family peptidase)	A0A6V7DZR4	*Xanthomonas hortorum* pv. *vitians*	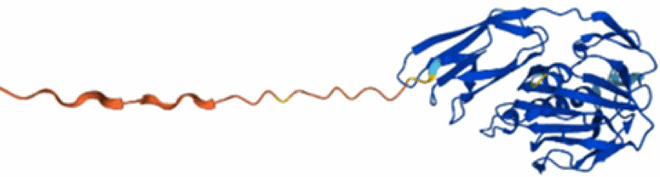
Xanthomonin I lasso peptide (Rosenthal et al. 2022)	F0CAT0	*Xanthomonas hortorum* ATCC 19865	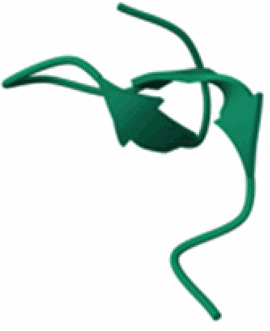 PDB:4NAG
Xanthomonin II lasso peptide (Rosenthal et al. 2022)	F0CAT1	*Xanthomonas hortorum* pv. *vitians*	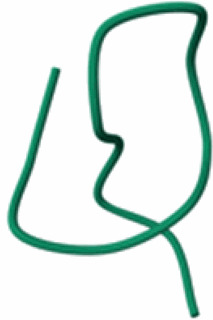 PDB: 2MFV
Enterocin lasso protein (Rosenthal et al. 2022)	LR828253.1: 2648847- 2648599 Similar to A0A1E3H528	*Xanthomonas hortorum* pv. *vitians*	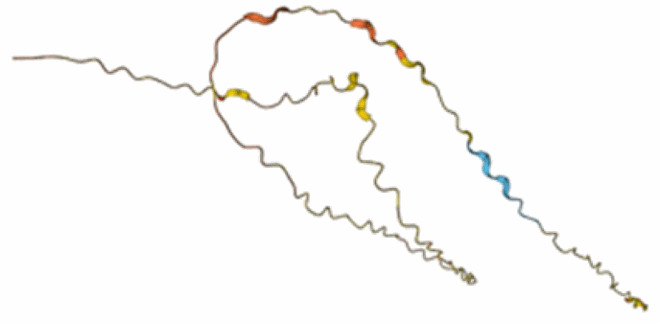 A0A1E3H528
Lectin-like LlpA_Xcm761_ (Ghequire et al. 2012)	Q8PP20	*Xanthomonas citri* pv. *malvacearum*	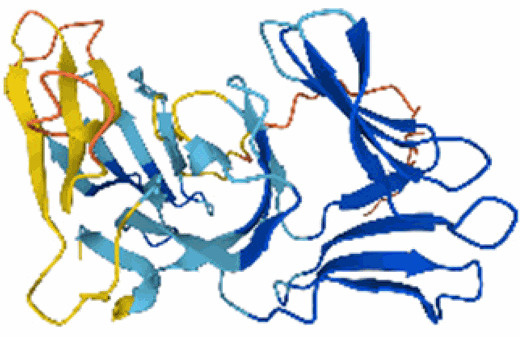
Lipid II-degrading bacteriocin (Grinter and Walker 2014)	A0A8A4W4D3	*Xanthomonas citri* pv. *fuscans*	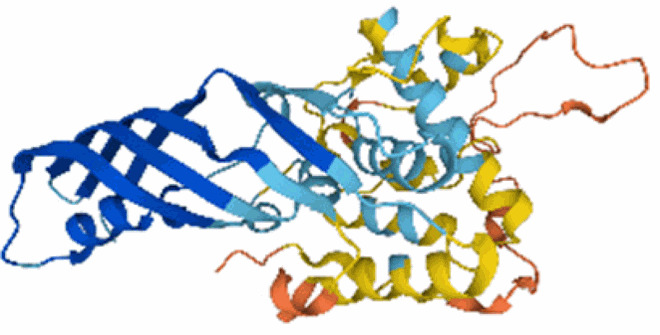

Several authors have pointed out that bioinformatics scans of *Xanthomonas* genome sequences reveal genes that encode likely bacteriocin products (Holtsmark et al. 2008; Salzberg et al. 2008; Sharp et al. 2017; Ghequire et al. 2018; Rooney et al. 2020a; Rosenthal et al. 2022). Colicin-like genes were reported in genome sequences of *X. oryzae* pv. *oryzae* and some RTX toxins that are distributed widely among Gram-negatives and may have antimicrobial activity (Holtsmark et al. 2008). *Xanthomonas* genomes also encode homologues of lipid-II-degrading bacteriocins (Grinter et al. 2012; Grinter and Walker 2014).

The most comprehensive attempt at mining *Xanthomonas* genomes for bacteriocins appears in a review published in 2008, when only a handful of genome sequences were available (Holtsmark et al. 2008). This study used BAGEL, a bacteriocin-mining tool that predicts bacteriocin ORFs from (meta-) genomic DNA sequences (de Jong et al. 2006). BAGEL identified between 5 and 10 peptide bacteriocins encoded in each available genome of *X. campestris* pv. *campestris*, *X. citri* pv. *citri*, *X. euvesicatoria* and *X. oryzae* pv. *oryzae*. However, the authors did not provide sequences, accession numbers nor genomic locations for these putative bacteriocins.

A more recent genome mining is presented in a comparative genomics study focussed on the lettuce pathogen *X. hortorum* pv. *vitians* (Rosenthal et al. 2022). That study used BAGEL 4 (van Heel et al. 2018) scanned genomes of *X. euvesicatoria* pv. *coriandri* as well several pathovars of *X. hortorum*. This identified genes encoding lanthipeptides and sactipeptides that may or may not be bacteriocins as well as lasso peptides, enterocins and the bacteriolysin zoocin A. Specific details of the putative bacteriocin genes were not provided. By repeating BAGEL4 searches against these genomes, we confirmed the presence of genes for lasso peptides xanthomonin I and II, zoocin A and enterocins (Table 3). Our BAGEL4 searches of *Xanthomonas* genomes also confirmed the presence of numerous open-reading frames with coding potential for other small microcin-like peptides. However, based on BLAST and BAGEL4 searches, we did not find close homologues of rhodanodin (Hegemann et al. 2015) in *X. hortorum* genomes. The most similar *Xanthomonas* sequence matches to rhodanodin are xanthomonins I/II and predicted proteins annotated as ‘benenodin family’ (Zong et al. 2017) (e.g. UniProt: A0A6V7BB03).

Many small bacteriocin-encoding ORFs in *Xanthomonas* do not appear as predicted protein-coding genes in the protein databases such as UniProt Knowledgebase (Magrane and UniProt Consortium 2011) and therefore are not represented in the AlphaFold (Jumper et al. 2021) database of predicted structures. This underscores the importance of performing genome mining at the level of DNA sequence rather than on predicted proteomes. Where available, predicted structures should be treated with caution (Terwilliger et al. 2024). For a few peptides, experimentally determined 3D structures are available as well as AlphaFold predictions, for example xanthomonins (Hegemann et al. 2014). For these proteins, the AlphaFold predictions fail to accurately capture the characteristic lasso structure that is evident in the NMR-determined structures (deposited in PDB under accessions 2MFV and 4NAG).

### Strengths and challenges

The use of bacteriocins to target bacterial pathogens of crops has several strengths and challenges (Table 4).

**Table 4.** The strengths and challenges of using bacteriocins to control bacterial plant diseases.

Strengths	Challenges
Narrow host range for precision targeting of pathogens with minimal damage to the wider microbial community	Narrow host range may require use of multiple bacteriocins to be effective against disease caused by non-clonal pathogens
Some bacteriocins have already gained FDA approval for use as food preservatives and have been deemed safe for human consumption	Need rigorous safety and environmental testing before regulatory approval
Can be applied as curative and preventative treatments for pre- and post-harvest diseases	Research required to develop application techniques and understand stability in the field
Mechanistically distinct bacteriocins can be combined and applied as a cocktail to reduce likelihood of resistant bacteria emerging	Emergence of bacteriocin-resistant pathogens
The sequencing era has provided a wealth of publicly available bacterial genome sequences to mine for new bacteriocins	Can be toxic in traditional *E. coli* production systems

*Precision targeting*: Unlike traditional chemical treatments (copper and antibiotics), most bacteriocins have a narrow host range (Heilbronner et al. 2021) and so could be used to specifically target plant pathogenic *Xanthomonas* while ensuring minimal damage to the wider microbial community and environment. However, some bacteriocins can have broad-range antimicrobial activity, e.g. fermenticin and nisin (Darbandi et al. 2022), so it is imperative that any prospective bacteriocins against *Xanthomonas* pathogens be tested against microbiomes (rhizosphere and phyllosphere) to identify any unintended targets that may negatively impact the plants’ fitness.

*Genome sequences available*: As illustrated in Table 3, candidate bacteriocins can be identified in complete and draft *Xanthomonas* genome sequences, of which there are many, using several complementary approaches: homology searches against known bacteriocins, e.g. BLAST (Altschul et al. 1990) and BAGEL (de Jong et al. 2006), metabolite genome mining pipelines such as antiSMASH (Blin et al. 2023) and machine-learning methods that do not rely on homology (Hamid and Friedberg 2019). Once identified, bacteriocins can be deposited into appropriate public databases like BACTIBASE for bacteriocins produced by Gram-positive bacteria (Hammami et al. 2007), to facilitate collaborative research and development of commercial bacteriocin products.

*Regulatory approval*: Some bacteriocins are already approved by the FDA as food preservatives (Schofs et al. 2020; Naskar and Kim 2021). Thus, they have been deemed safe for human consumption, and it should therefore be reasonable to assume that bacteriocins could be applied to food crops in the field, although rigorous safety and environmental testing would be essential. Moreover, their prior approval as food preservatives may mean that the use of bacteriocins as plant protection products could be better received by the public compared to that of crops that have been genetically modified/edited for resistance to *Xanthomonas* diseases.

*Production*: One likely challenge is the difficulty in producing some bacteriocins in traditional *E. coli* protein expression systems due to toxicity issues. However, alternative methods are available, such as using *Spodoptera frugiperda* (Sf9) insect cells (Girardin et al. 2024).

*Application*: Traditionally, *Xanthomonas* diseases have been controlled using preventative measures but bacteriocins have the added advantage that they could be applied as a preventative as well as a curative control for both pre- and post-harvest diseases. This is important, as xanthomonads have been associated with post-harvest soft rots of many horticultural crops, like brassicas, which can lead to total crop losses (Anand and Lata D 2021; Greer et al. 2023). However, there are several knowledge gaps regarding bacteriocin application that need to be addressed, including the stability of bacteriocins in the field, application method (e.g. spray or seed treatment), application timing, whether they should be applied as a (semi-) pure product or as an inoculation with the producing bacteria and whether they would be compatible with other biocontrol methods like beneficial microbes (Marin et al. 2019).

While there remains public and regulatory resistance to genetically modified plants, genetic engineering could also be used to intelligently deploy bacteriocins to protect from *Xanthomonas* infection. For example, *Xcc* infects its brassica hosts primarily through the hydathodes, from where it then moves through the vasculature and multiplies (Luneau et al. 2022). *Xcc* moves systemically to the seed and is a major agricultural challenge, as it is a key mode of disease transmission globally (CABI 2022). Thus, potential to target bacteriocin expression to the hydathodes, vasculature or even the developing or germinating seed provide exciting opportunities to develop *Xcc*-resistant *Brassica* crops.

*Bacteriocin-resistant xanthomonads*: To effectively exploit bacteriocins in the field and mitigate the emergence of bacteriocin-resistant strains, it will most likely be necessary to deploy two or more bacteriocins that target different receptors/uptake pathways. Bacteria can rapidly evolve and adapt to their changing environments. They have been shown to readily acquire resistance genes to antibiotics via transformation-, conjugation- and transduction-mediated horizontal gene transfer (HGT) (Arnold et al. 2022) but there is scarce information on how they might acquire resistance to bacteriocins. Some studies have shown that mutations in bacteriocin cell-surface receptors and certain environmental conditions can result in resistance (Gradisteanu Pircalabioru et al. 2021).

## Realising the untapped potential for dual phage and bacteriocin targeting of key bacterial diseases

**Figure 3.**
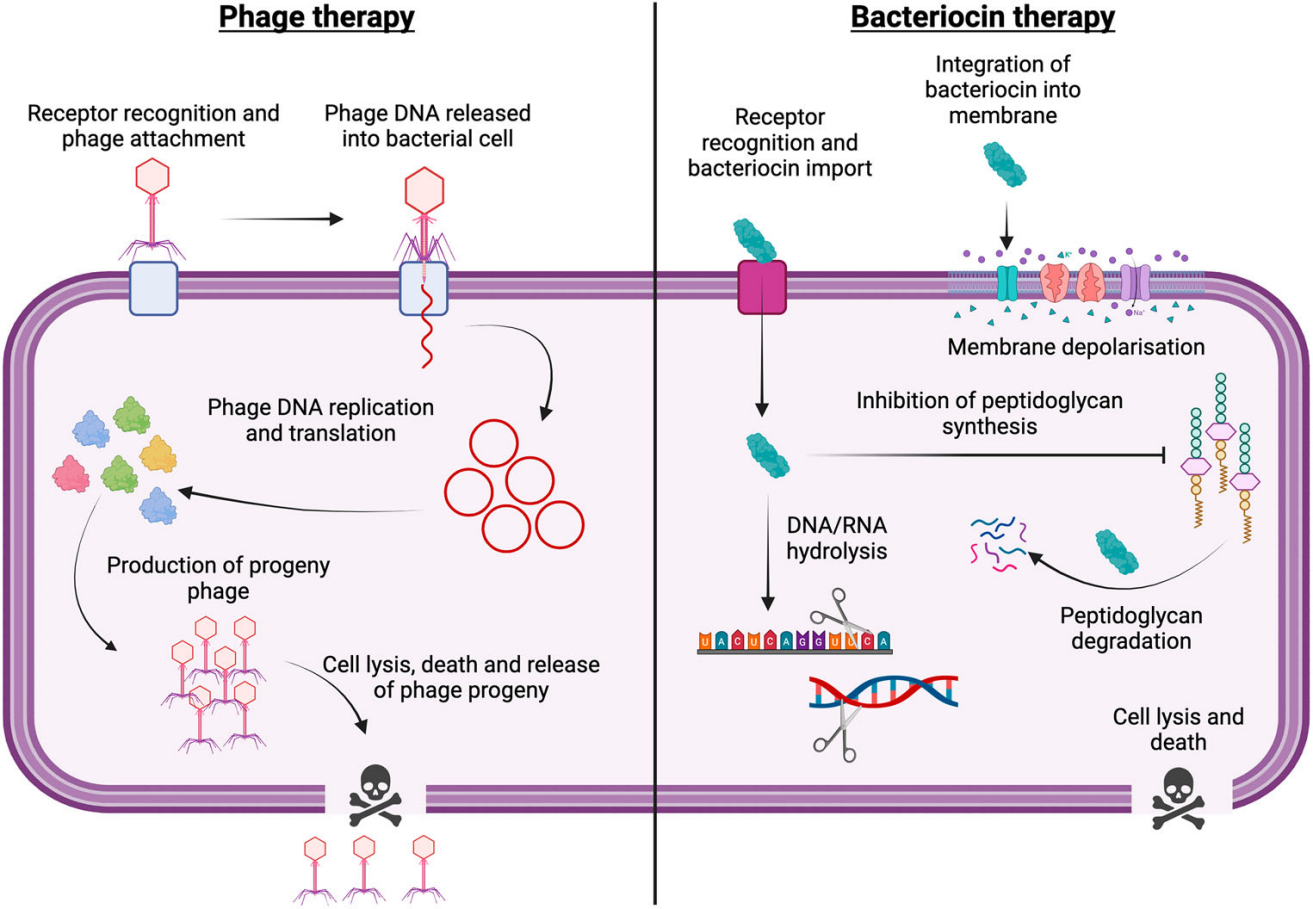
Bactericidal mechanisms of lytic bacteriophages and bacteriocins. Created in Biorender.com.

The key objective of this review is to present alternative strategies that may be more effective in managing phytobacterial diseases. The simultaneous deployment of phages and bacteriocins, thus ‘attacking’ the phytopathogen from two fronts, may be an effective method of reducing evolution of resistance against phages or specific bacteriocins. Because these two antibacterial approaches rely on activation of mechanistically distinct processes (Figure 3), this would impose an additional load on bacterial fitness and constrain evolution of bacterial fitness. Such an approach could, if economically viable, also be combinatorial, with co-delivery of phage cocktails with mechanistically distinct bacteriocins (different pathways and/or receptors). This could allow broad-spectrum protection against diverse isolates of a given *Xanthomonas* pathogen. For example, there are multiple prevalent races of the black rot pathogen *Xcc* that infect brassicas (Vicente et al. 2001). Phage-bacteriocin cocktails could be used in place of environmentally damaging chemical treatments and in combination with varietal resistance to *Xcc* in brassicas, which typically are only effective against specific races (Vicente et al. 2002). The use of phage-bacteriocin cocktails in conjunction with varietal resistance would provide a third layer of plant protection and could prevent/reduce the likelihood of varietal resistance-breaking.

Despite several knowledge gaps, bacteriocins and phages remain a promising approach for the biocontrol of bacterial pathogens like xanthomonads. Further research is needed to better understand persistence and mode of action as well as exploring how bacteriocins can be used in combination with phage biocontrol to manage bacterial phytopathogens effectively and sustainably or indeed whether phage/bacteriocins cocktails are compatible. In this context, it would be important to ascertain whether (i) bacteriocins are compatible with phage formulations, preservation and application methodology, and (ii) if phage biocontrol may encourage transduction/prophage-mediated gene transfer of bacteriocin immunity genes or mutations leading to bacteriocin resistance. However, it is best practise not to use transducing phages as biocontrols for such reasons.

## Summary

Here we propose that, as our knowledge expands and a more genomics-informed bacteriocin selection pipeline can be implemented, combinatorial application of bacteriocins and phages represents a novel phytobacterial disease control option. It provides an exciting avenue for innovative, targeted and potentially more environmentally friendly agricultural practices. Using *Xanthomonas* diseases as an exemplar, we have shown the potential synergy between bacteriocins and phages. The combination of both offers dual and precise targeting with agents that have distinct intervention strategies, reducing the collateral damage often associated with broad-spectrum chemical treatments. Bacteriocin-phage cocktails would be anticipated to be synergistic to some extent, as well as making it more difficult for bacteria to evolve resistance as they try to mitigate two different antibacterial strategies targeting different molecular pathways. Moreover, utilising natural agents like bacteriocins and phages is not only likely to have fewer detrimental effects on the environment compared to synthetic chemical pesticides, but also be more publicly acceptable. Developing a comprehensive understanding of how bacteriocins and phages interact, their optimal combinations and their application methods still require significant research. However, the benefits as well as the knowledge accrued are potentially invaluable for managing phytobacterial diseases, though implementation of robust regulatory frameworks will be essential to harness their full potential.

## Author contributions

MG conceptualised the manuscript. All authors were involved with the writing of the original draft, reviewing and editing. All authors read and approved the submitted version of the article.
